# Social Statistics Transformation: Understanding the population through the production of income by ethnicity statistics from administrative data

**DOI:** 10.23889/ijpds.v8i2.2243

**Published:** 2023-09-14

**Authors:** Joanna Harkrader, Michelle Bellham, Samantha Pendleton, Alison Morgan, Joe Pearce, Emily Stennard

**Affiliations:** 1 Welsh Government, Cardiff, United Kingdom

## Objectives

The lack of an income question on the Census has meant the production of multivariate income by ethnicity statistics has not been possible in census outputs to date. Our ambition is to provide individual-level records for every member of the usually resident population of England and Wales using admin data.

## Methods

Exciting progress has been made in the development of admin-based characteristics measures, including the ongoing feasibility research to produce admin-based datasets on ethnic group and income. Demonstrated improved results include the coverage of the population steadily increasing and methods to create these datasets gradually improving.

Access to these record-level administrative datasets has allowed us to combine admin-based income and ethnicity measures developed in previous research, linking individuals between the two. We review the coverage of the combined dataset and the feasibility of producing multivariate statistics at subnational levels in England and Wales for the first time.

## Results

This presentation will showcase our innovative progress so far. By combining admin-based income and admin-based ethnicity datasets, we established an income and a stated ethnicity for 77.1% of people in England and 82.1% of people in Wales aged 16 years and over in the admin-based Statistical Population Dataset (our population base).

For the first time, we have produced income percentiles for ethnic groups at different levels of geography in England and Wales including national figures, regional figures and figures for local authorities and lower layer super output areas; although statistical disclosure control means that some of the figures have been suppressed.

We will highlight some of the challenges in using administrative data sources to produce these statistics and in assessing their statistical quality.

## Conclusion

Our research developing these statistics is truly novel and shows much promise. Future work will include research to improve the univariate admin-based measures that are used, to continue to explore the limitations of the combined dataset, to explore the data by occupied address, and explore methods to adjust for missingness.

